# A pragmatic randomized control trial and realist evaluation on the implementation and effectiveness of an internet application to support self-management among individuals seeking specialized mental health care: a study protocol

**DOI:** 10.1186/s12888-016-1057-5

**Published:** 2016-10-18

**Authors:** Jennifer M. Hensel, Jay Shaw, Lianne Jeffs, Noah M. Ivers, Laura Desveaux, Ashley Cohen, Payal Agarwal, Walter P. Wodchis, Joshua Tepper, Darren Larsen, Anita McGahan, Peter Cram, Geetha Mukerji, Muhammad Mamdani, Rebecca Yang, Ivy Wong, Nike Onabajo, Trevor Jamieson, R. Sacha Bhatia

**Affiliations:** 1 Women’s College Hospital Institute for Health Systems Solutions and Virtual Care, Women’s College Hospital, 76 Grenville St, Toronto, ON Canada; 2 Department of Psychiatry, Women’s College Hospital and University of Toronto, 76 Grenville St, Toronto, ON Canada; 3 Women’s College Research Institute, Women’s College Hospital, 76 Grenville St, Toronto, ON Canada; 4 Li Ka Shing Knowledge Institute, St. Michael’s Hospital, 209 Victoria St, Toronto, ON Canada; 5 Department of Family and Community Medicine, Women’s College Hospital and University of Toronto, 76 Grenville St, Toronto, ON Canada; 6 Institute for Health Policy, Management and Evaluation, University of Toronto, 155 College St, Toronto, ON Canada; 7 Institute for Clinical Evaluative Sciences, 2075 Bayview Ave, Toronto, ON Canada; 8 Department of Family and Community Medicine, University of Toronto, 500 University Ave, Toronto, ON Canada; 9 Women’s College Hospital Family Health Centre, 77 Grenville St, Toronto, ON Canada; 10 OntarioMD, 150 Bloor St, Toronto, ON Canada; 11 Rotman School of Management, University of Toronto, 105 St. George St, Toronto, ON Canada; 12 Division of General Internal Medicine and Geriatrics, University Health Network and Sinai Health System, and University of Toronto, 600 University Ave, Toronto, ON Canada; 13 Department of Medicine, University of Toronto, 1 King’s College Circle #3172, Toronto, ON Canada; 14 Li Ka Shing Centre for Healthcare Analytics Research and Training, St. Michael’s Hospital, 209 Victoria St, Toronto, ON Canada; 15 Leslie Dan Faculty of Pharmacy, University of Toronto, 144 College St, Toronto, ON Canada

**Keywords:** Web-based, Internet, Virtual care, Implementation, Self-management, Recovery

## Abstract

**Background:**

Mental illness is a substantial and rising contributor to the global burden of disease. Access to and utilization of mental health care, however, is limited by structural barriers such as specialist availability, time, out-of-pocket costs, and attitudinal barriers including stigma. Innovative solutions like virtual care are rapidly entering the health care domain. The advancement and adoption of virtual care for mental health, however, often occurs in the absence of rigorous evaluation and adequate planning for sustainability and spread.

**Methods:**

A pragmatic randomized controlled trial with a nested comparative effectiveness arm, and concurrent realist process evaluation to examine acceptability, effectiveness, and cost-effectiveness of the Big White Wall (BWW) online platform for mental health self-management and peer support among individuals aged 16 and older who are accessing mental health services in Ontario, Canada. Participants will be randomized to 3 months of BWW or treatment as usual. At the end of the 3 months, participants in the intervention group will have the opportunity to opt-in to an intervention extension arm. Those who opt-in will be randomized to receive an additional 3 months of BWW or no additional intervention. The primary outcome is recovery at 3 months as measured by the Recovery Assessment Scale-revised (RAS-r). Secondary outcomes include symptoms of depression and anxiety measured with the Personal Health Questionnaire-9 item (PHQ-9) and the Generalized Anxiety Disorder Questionnaire-7 item (GAD-7) respectively, quality of life measured with the EQ-5D-5L, and community integration assessed with the Community Integration Questionnaire. Cost-effectiveness evaluations will account for the cost of the intervention and direct health care costs. Qualitative interviews with participants and stakeholders will be conducted throughout.

**Discussion:**

Understanding the impact of virtual strategies, such as BWW, on patient outcomes and experience, and health system costs is essential for informing whether and how health system decision-makers can support these strategies system-wide. This requires clear evidence of effectiveness and an understanding of how the intervention works, for whom, and under what circumstances. This study will produce such effectiveness data for BWW, while simultaneously exploring the characteristics and experiences of users for whom this and similar online interventions could be helpful.

**Trial registration:**

Clinicaltrials.gov NCT02896894. Registered on 31 August 2016 (retrospectively registered).

## Background

Mental illness is a substantial and rising contributor to the global burden of disease [1]. In Canada, 1 in 5 people are affected by this leading cause of disability, associated with more than $51 billion CAD in annual costs [2]. Utilization of mental health care, however, is limited by structural barriers such as specialist availability, geography, time, out-of-pocket costs for patients [3, 4], and attitudinal barriers including stigma [4]. It is estimated that under-recognition and/or stigma act as obstacles to accessing care for at least 50 % of those affected [4–6].

Innovative solutions such as virtual care, broadly defined as any remote interaction between patients and/or health care providers using any form of information technology to enhance healthcare [7, 8], are rapidly entering the health care domain. While these innovative solutions may address some mental health care access issues through components such as anonymity and rapid availability, their advancement and adoption often occurs in the absence of rigorous evaluation and adequate planning for sustainability and spread [9].

Studies evaluating virtual mental health applications have demonstrated conflicting findings, with some trials demonstrating no benefits [10] and others demonstrating substantial improvement among participants [11]. These conflicting findings illustrate the importance of studying the processes of implementation, including contextual factors, and the mechanisms of action that help to determine whether and how virtual mental health applications have effects on patient outcomes [12]. Although a wide variety of theories and methods are available to focus attention on key issues in the adoption and scale-up of new technologies, these are only recently being applied in large implementation studies [13, 14].

In the province of Ontario, Canada, the Ontario Telemedicine Network (OTN), a non-profit and government-funded organization, is the largest provider of telemedicine services [15]. In 2015, OTN conducted a selection process to initiate a series of pilot telehomecare interventions for patients with chronic conditions, ultimately choosing to test Big White Wall (BWW) for those struggling with mental health. This manuscript describes the approach to the evaluation of this virtual mental health application within the local health care system in Ontario, Canada.

### Virtual care application: Big white wall

BWW is an internet-based application, built on evidence-based components of mental health care (eg. peer support [16], cognitive behavioural therapy [17]) combined in a virtual environment that provides anonymity (see www.bigwhitewall.com). BWW offers users access to self-assessment tools, a social network that connects people who have similar problems, and access to on-line courses. The service is monitored at all times by “Wall Guides”, trained mental health professionals (psychiatrists, social workers, and psychologists), ensuring that users are responded to and that the content posted is appropriate and safe. BWW was first developed and implemented within the National Health Service in the UK in 2007, and is now available in the UK, New Zealand, and the USA with over 35,000 users since its inception [18]. An independent pre-post evaluation of BWW users in the UK over 3 months found that 50 % showed a meaningful reduction in symptoms using validated scales of depression and anxiety [18]. While more rigorous evaluations of BWW are currently underway, it has not yet been comprehensively evaluated, and not within a Canadian context.

This protocol outlines a mixed methods study guided by a theoretical approach to examine the implementation of BWW in Ontario. The overall approach taken in this study employs a novel model of integrated knowledge translation [19], whereby the health system decision makers, implementation leads, and scientific evaluators have been engaged in the development of the research project from the outset. This approach facilitates the assessment of implementation and scalability, and increases the likelihood that results will have a future impact on health system policy.

## Methods/design

### Study aims

This study aims to evaluate whether the intervention, BWW, is acceptable, effective and cost-effective. In addition, the study aims to generate knowledge on the implementation processes and outcomes that will inform future health system decision-making regarding the potential scale-up of this and other mental health virtual care applications in Ontario.

### Hypotheses

We hypothesize that access to BWW will increase mental health recovery orientation and reduce symptoms of depression and anxiety among patients seeking specialized mental health care. Further, we expect that a greater response to BWW will be predicted by higher baseline belief in credibility and outcome expectancy of the intervention as well as actual level of engagement with the intervention. The core mechanisms by which we hypothesize BWW to be effective include: 1) enhanced self-efficacy to self-manage mental health conditions through acquisition of knowledge, personal use of tracking tools, and vicarious learning; and 2) rapid access to peer and facilitated support within a constrained health care system leading to a feeling of security and enhanced ability to cope in crisis situations.

### Study setting

This study is based in Ontario, Canada’s most populous province. In Ontario, all physician and hospital mental health care, including outpatient services located in hospitals, are publicly funded under the single-payer provincial health insurance plan [20]. Subsidized community services offer some services for free or at a low cost. [20] There is also a large network of private inter-professional health providers including social workers, psychologists and registered psychotherapists who provide counselling and psychotherapy for a fee [21]. Third party insurers may offer partial or limited coverage for these services [22]. Due to limited publicly funded services and high costs for private services, public programs have long wait times on the order of months to a year or more, and often provide time-limited treatment only [23, 24]. Although the potential for virtual mental health care platforms within this resource constrained system is apparent, few widely accessible platforms exist, and none with sufficient evidence to warrant their wide scale adoption into clinical practice.

Within Ontario, three test sites were selected from cities with populations of approximately 80,000, 150,000, and 2.5 million. These sites include: 1) a community general hospital with inpatient and outpatient mental health services and an emergency department (ED) that sees a high volume of mental health-related visits, 2) a psychiatric hospital with inpatient and outpatient mental health services, and 3) an academic ambulatory care hospital with a large outpatient mental health program. With the exception of the ED, the majority of the clinical programs accept referrals from the community or from within their respective hospital for assessment and management of chronic and/or treatment refractory mental health problems. The third site offers self-referral to some of the treatment programs with the requirement that individuals undergo an extensive intake assessment prior to being formally accepted to the waitlist.

### Participant eligibility

Eligible patients are individuals 16 years of age and older who are seeking mental health care at any of the participating sites. Inclusion criteria: 1) able to provide informed consent; 2) able to read in English; 3) able and willing to access the internet, 4) able to navigate an on-line program independently or with minimal assistance; and 5) have or are willing to obtain and use an email address. Exclusion criteria: There are no exclusion criteria but clinicians are reminded that BWW is a peer community and participants need to be able to coherently and respectfully interact with others. As such, we do not recommend individuals with severe psychosis or behavioural disorders be recruited.

### Evaluation framework

**Fig. 1:**
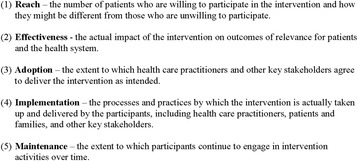
The RE-AIM Framework

The RE-AIM framework [25] will guide the study of whether and how to scale BWW across Ontario. RE-AIM draws researchers’ attention to the issues of Reach, Effectiveness, Adoption, Implementation, and Maintenance (see Fig. 1). Within this framework, the RCT aims to evaluate reach and effectiveness, and Realist Evaluation will explore adoption, implementation and maintenance. Realist Evaluation [26] is growing in international popularity in the health services and policy research community [27], and is proposed for the evaluation of complex health care interventions [26]. Realist Evaluation has a number of appealing features for researchers and health system decision makers, as it does not simply provide a binary indication of whether an intervention works or not. Instead, it answers the question, “what works, for whom, under what circumstances” [28]?

### Study design

**Fig. 2:**
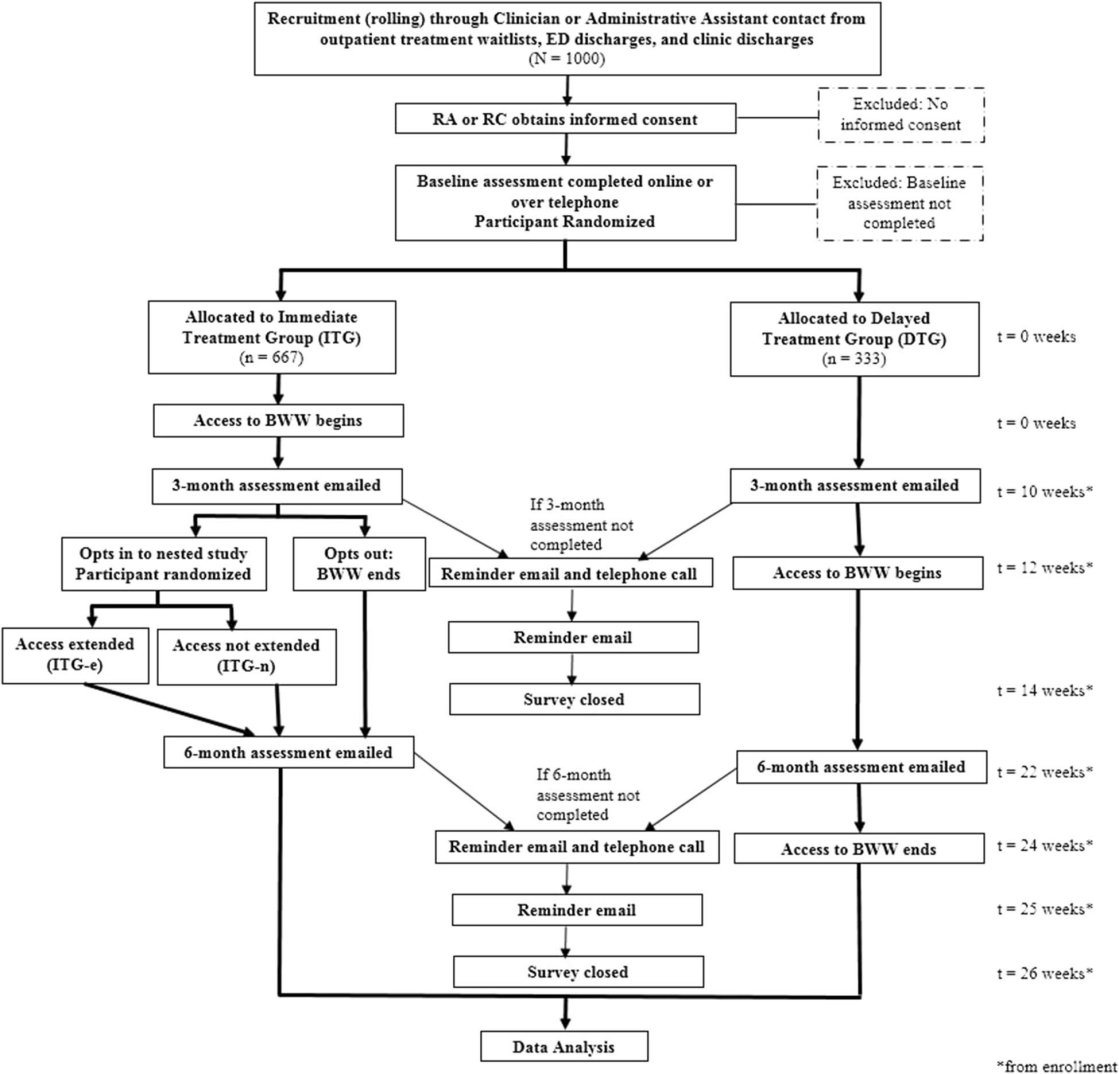
Detailed study flow for the RCT. ED: Emergency Department; RA: research assistant; RC: research co-ordinator; BWW: Big White Wall

This study consists of a pragmatic randomized controlled trial (RCT) with a nested comparative effectiveness arm to assess the value of intervention extension, with concurrent Realist Evaluation. At enrollment, participants will be randomized to either immediately receive the intervention, BWW (immediate treatment group; ITG) or receive access to BWW after a 3 month waiting period (delayed treatment group; DTG). The DTG will serve as the control group during the first 3 months. After 3 months of intervention, ITG participants will have the opportunity to opt-in to an intervention extension arm. Those who opt-in will be randomized to receive an additional 3 months of BWW or no additional intervention. A purposefully sampled sub-group of trial participants, health care providers, and health system decision makers will be invited to participate in qualitative interviews throughout. The RCT study flow is depicted in Fig. 2.

### Part 1. Pragmatic randomized controlled trial

#### Recruitment

Participants will be recruited at a number of entry or exit points including outpatient clinics and their waitlists, emergency department (ED) discharges, and rapid assessment clinic discharges. Outpatient clinics include youth mental health services (age 16 and over only), adult general psychiatry consultation services (typically serving mood and anxiety disorders), psychotherapy services, post-traumatic stress and trauma therapy services, an outpatient substance use program, and a borderline personality disorder clinic. These recruitment settings were selected because: 1) they serve patients with the potential to benefit from the intervention, 2) they were identified by the participating sites and OTN as high priority settings, and 3) they have high volumes of use and turnover that will allow the implementation organization to reach their recruitment target. With the exception of the ED and rapid assessment clinics, the wait times to access these services vary from 4 months up to 2 years. Participation in the study will not affect wait list position or access to services.

Potential participants will be approached by a member of the clinical staff either by telephone, or in person in the clinic or ED. Hard-copy pamphlets or a website link to the study information will be provided. Once a potential participant indicates their interest, a research assistant (RA) or research co-ordinator (RC) will either meet them in person or contact via telephone to provide more information and obtain consent. Consent will include: 1) Consent to participate in the RCT (required), 2) Consent to be contacted for a qualitative interview (optional), 3) Consent to provide individual provincial health insurance plan (OHIP) number for linkage of survey data to health administrative data (optional), and 4) Consent for primary care provider to be notified of study enrollment (optional).

Separate consent to be re-randomized at 3 months will be obtained from ITG participants who opt-in to the nested intervention extension arm of the trial. This will be done with a question posed at the end of the 3 month data collection survey.

#### Allocation and blinding

At enrollment, a 2:1 allocation ratio will be used to randomize participants to ITG or DTG. This allocation ratio was chosen to immediately provide the intervention to more participants and increase likelihood of participation. Participants and providers will not be blind to treatment group. Randomization sequences will be generated independently and will be stratified by site and recruitment setting. Allocation sequence will be concealed until after baseline data has been collected to blind those involved in recruiting and consenting procedures. Group allocation will be revealed by phone or email. At t = 3 months, ITG participants who opt in to the nested study arm, will be randomized 1:1 to a 3 month BWW extension (ITG-e) or no extension (ITG-n). Group allocation will again be revealed via phone or email. The nested treatment extension arm will be concealed from participants at enrollment to encourage maximal usage of the intervention during the first 3 months. All data analyses will be blinded.

#### Interventions

##### Treatment as usual (months 0–3 for DTG)

Participants in the DTG will receive treatment as usual with no intervention from the study for the first 3 months. Treatment as usual may include formal or informal mental health care including pharmacological and non-pharmacological treatments. Participants recruited at discharge from clinics or the ED will receive usual recommendations regarding other services and programs that would routinely be offered upon discharge from those settings.

##### Treatment as usual + BWW (months 0–3 for ITG, months 4–6 for DTG and ITG-e)

Participants will receive a 3-month subscription to BWW, free of charge. Use of BWW is participant-driven, with the ability to utilize the services at any frequency. A unique BWW access code will be emailed to participants. If registration has not occurred within 3 days of receipt, a follow-up call will be made to remind the participant to log in. For the DTG participants, a study team member will contact them 3 months after enrollment to remind them that their access to BWW will start ﻿and the access code will be emailed. Participants randomized to ITG-e will be able to reactivate their same account once allocation has been revealed.

##### Participant safety on BWW

All participants maintain anonymity on the site through a unique non-identifiable user ID. Study participants will have a unique landing page that includes contact information for local crisis services in the event urgent assistance is required. BWW uses automated word recognition software that will alert the Wall Guides when potential safety issues are arising (eg. a user mentions ‘suicide’). The Wall Guides will then monitor that user’s activity and if deemed clinically necessary, will intervene to offer support. In the event that urgent support is required, they will direct them to use the crisis services listed on the landing page.

#### Data collection

All baseline and follow-up data will be entered into a REDCap™ database developed for the RCT. REDCap™ is confidential and is only accessible to the study personnel by secure login.

##### Baseline data

At time of consent, participants will complete a baseline questionnaire assessing socio-demographics, mental health history, measures of treatment credibility and outcome expectancy, and all outcome measures. Due to the length of time required to complete consent and all baseline questionnaires, baseline data may be collected in 1 of 3 ways depending on recruitment setting and participant preference: 1) by web survey (default); 2) by phone; or 3) in-person hard-copy.

##### Socio-demographics

Baseline data will include participant age, gender, ethnicity, education, relationship status, household income, employment status, and living situation.

##### Mental health history

At baseline, we will collect age at first onset of mental health problems, age at which formal mental health support was first sought, and duration of current episode.

##### Baseline belief in treatment credibility and outcome expectancy

Participants are asked to rate their belief in the credibility of self-help resources to improve mental health with the author-generated statement: “Self-help tools including on-line services or books are helpful for people with mental health problems” on a 4-point scale from ‘definitely agree’ to ‘completely disagree.’ In addition, they will be asked about their outcome expectancy of BWW to improve their mental health. Item #4 from the Credibility and Expectancy Questionnaire [29] will be used, “After having access to BWW for 3 months, how much improvement in your mental health do you think will occur?” Participants are asked to rate their response from 0 to 100 % with options available in 10 % increments. This single item has been shown to correlate strongly with psychotherapy outcomes [30].

##### Follow-up data (3 and 6 Months)

REDCap™ will be automated to push web-based surveys containing all outcome measures to participant emails 2 weeks prior to each pre-determined data collection time point. If, after 2 weeks, the participant has not completed the survey, a follow-up phone call will be made and confirmation of intention to complete it will be obtained and the link pushed again, or the questionnaire will be completed over the phone. For the former option, after 1 week, if still not complete a second follow-up phone call will be made and every effort to collect data over the phone will be made. All outcome measures must be completed within ±2 weeks of the intended time point.

##### BWW utilization data

BWW will provide a report of each participants’ use at 2 time points during the study: 1) after all participants have been enrolled for 3 months, and 2) study end. This will include total number of logins, average and total time on the site, along with logs of activity such as pages viewed and posts made.

##### BWW satisfaction

At the end of the BWW access period (month 3 for ITG, month 6 for DTG) participants will be asked to rate BWW overall and indicate if they would recommend it, pay for it, and continue to use it if available.

#### Outcome measures

##### Primary and secondary outcomes

The primary outcome is the total score on the Recovery Assessment Scale-revised (RAS-r) at 3 months. The RAS-r is a 24-item validated pan-diagnostic consumer-oriented outcome measure [31]. All items are scored on a 5-point scale from ‘strongly disagree’ to ‘strongly agree.’ The choice of the RAS-r reflects the current “recovery era” for mental health policy and services and the increasing focus on consumer achievement of satisfying and fulfilling lives, rather than being symptom free [31]. Secondary outcomes include the five subscale scores of the RAS-r: 1) personal confidence and hope, 2) willingness to ask for help, 3) goal and success orientation, 4) reliance on others, and 5) not dominated by symptoms; symptoms of depression and anxiety measured with the Personal Health Questionnaire-9 item (PHQ-9) and the Generalized Anxiety Disorder Questionnaire-7 item (GAD-7) respectively; quality of life measured with the EuroQOL group’s EQ-5D-5 L [32]; and community integration assessed with the Community Integration Questionnaire (CIQ) [33]. The 15-item CIQ is a brief, reliable measure of a person’s level of integration into the home and community. The overall score ranges from 0 to 29 with a higher score indicating better integration. The CIQ can also be divided into 3 subscales: 1) home integration, 2) social integration, and 3) productivity [33]. Timing of the completion of outcome assessments is outlined in Table 1.

**Table 1:** Timing of outcome assessments

Measures/Assessments	Baseline	3 months	6 months
RAS-r	X	X	X
PHQ-9	X	X	X
GAD-7	X	X	X
CIQ	X	X	X
EQ-5D-5 L	X	X	X
Adapted CSRI^a^	X	X	X
Out-of-pocket costs^b^	X	X	X
Health Administrative Data	N/A	X	X
BWW satisfaction	N/A	X (ITG)	X (DTG)

*RAS-r* recovery assessment scale-revised, *PHQ-9* personal health questionnaire, 9-item, *GAD-7* generalized anxiety disorder questionnaire, 7-item, *CIQ* community integration questionnaire, *EQ-5D-5 L* Euro-QOL quality of life measure, *CSRI* client service receipt inventory, *BWW* big white wall, *ITG* immediate treatment group, *DTG* delayed treatment group, *N/A* not assessed

^a^Adapted for the Ontario health care context

^b^Includes costs for medication, laboratory investigations, mental health services, and physical health services

##### Health care utilization and costs

Self-reported health care utilization will be assessed with the Client Service Receipt Inventory [34], adapted for the Ontario context. This measure captures utilization of physician and hospital services as well as community mental health services. Health administrative data will be used to validate reports of physician and hospital care, however, community service use and non-physician visits are not captured in Ontario data. Studies of reliability of self-report data over a 3 month recall period demonstrate approximately 60 % exact match with roughly equal proportions of over and under-reporting [35]. High emotional distress may be associated with over-reporting, and hospital and ED use is typically more accurately self-reported than outpatient care [35]. The CSRI will also assess prescribed medication and adherence. Self-reported out-of-pocket costs for medication, laboratory investigations, mental health services, and physical health services will be captured using validated questions from the Commonwealth Fund Survey of patients with Complex Needs [36].

Health administrative data will be retrieved after study completion from the Institute for Clinical Evaluative Sciences (ICES), a not-for-profit research institute encompassing a secure and accessible array of Ontario's health-related data. At ICES, individual-level health care and socio-demographic data from various sources are de-coded and linked using an encrypted health care number (ICES Key Number). We will extract individual data on inpatient, ED, outpatient health care utilization, as well as prescription drug costs (for people on social assistance or over age 65) during the study period and link to individual survey data using encrypted OHIP identifiers created by ICES authorized personnel. Individual health care costs will be determined using methods developed for use with Ontario data [37].

We will track incremental costs associated with BWW using unit prices from expenditures related to its use (capital and operating costs such as license fees) as well as human resources required to support its implementation.

#### Adverse events

There is a growing consensus that adverse events (AEs) need to be evaluated in behavioural intervention trials [38, 39], as there is a theoretical risk of deterioration, especially if online self-help tools are misunderstood or not properly applied [38]. We will compare pre-defined AEs (see Table 2) between the ITG and DTG participants at 3 months, and the ITG-e and ITG-n participants at 6 months for clinically important differences that may be a result of BWW. Moreover, an independent data safety and monitoring board (DSMB) comprised of three content and methodology experts assembled for this trial will review available AE data at two interim time points: after the first 150 participants have completed 3 months, and again after 500 participants have completed 3 months, to determine if any investigation is required and if the trial is safe to continue. Health administrative data will not be available within this timeframe and will not be reviewed by the DSMB. DSMB terms of reference are available from the study investigators.

**Table 2:** Adverse Events

Adverse Event	Measure	Description
Mental health Hospitalization	CSRI; Health administrative data	Number of hospitalizations on a psychiatric unit
Mental health ED visit	CSRI; Health administrative data	Number of ED visits for a mental health reason
Crisis service use	CSRI	Number of times using crisis supports
Increased suicidal ideation	PHQ-9 item 9	Increase in PHQ-9 item 9 score
Death	Health administrative data	All-cause mortality
Worsening depression or anxiety	PHQ-9, GAD-7	Increase in PHQ-9 or GAD-7 score over the prior 3 months
Declining social and community integration	CIQ	Decrease in CIQ score over the prior 3 months
Worsening disability	EQ-5D-5 L	Decrease in EQ-5D-5L scores over the prior 3 months
Medication discontinuation	Author question added to CSRI	Self-reported discontinuation of psychotropic medication without provider knowledge

*CSRI* client service receipt inventory, *PHQ-9* personal health questionnaire, 9-item, *GAD-7* generalized anxiety disorder questionanire, 7-item, *CIQ* community integration questionnaire, *EQ-5D-5 L* Euro-QOL quality of life measure

#### Data analysis

In order to meet stakeholder deliverables, analyses will be completed at 2 time points: 1) analysis of 3 month data once all 3 month data have been collected, and 2) analysis of 6 month data at study end. Health care administrative data will only be analyzed after study end.

##### Primary and secondary analyses

Baseline descriptive statistics will be generated overall, between treatment groups and between recruitment settings. Employment status and medication use will be described at baseline and 3 months. Prior 3-month health care utilization assessed with the CSRI (and validated with health administrative data when available) will be described at baseline and at 3 months. All primary and secondary outcomes will be described at baseline and 3 months, including the RAS-r and CIQ subscales. In addition, at baseline and 3 months, we will describe the proportion of participants with PHQ-9 and GAD-7 scores of 10 or more, indicative of clinically important levels of depression and anxiety [40], respectively. At 3 months, we will describe clinically important cutoffs on the PHQ-9 and GAD-7 including a reduction of 5 or more points from baseline (clinically meaningful change), reduction in score of 50 % or more from baseline (response), and a score less than 10 (remission) [40]. Adverse event descriptive data will be reported at 3 months, and again at 6 months for the BWW extension group. BWW satisfaction descriptive data will be reported at 3 months for ITG participants and 6 months for DTG participants.

The primary outcome, RAS-r at 3 months, will be analyzed with an intent-to-treat analysis using an ANCOVA controlling for baseline RAS-r score as well as treatment group, unadjusted and adjusted for baseline PHQ-9, baseline GAD-7, age, sex, education, relationship status, household income, duration of episode, and recruitment setting. In sensitivity analysis, we will repeat this using a marginal structural model to account for attrition.

The same analysis will be repeated for all secondary outcomes at 3 months controlling for baseline score and treatment group. Analysis of 3 month data will be completed after all 3 month data have been collected.

In the subset of ITG participants who opt in to the nested extension study, we will examine outcomes at 6 months between treatment groups. Analysis of primary and secondary outcomes will be repeated as described above, controlling for scores at both baseline and 3 months.

##### Exploratory analyses

The first exploratory analysis will examine a subset of the ITG group who had a PHQ-9 or GAD-7 score of at least 10 at baseline. Participants will be categorized as ‘responders’ or ‘non-responders’ based on whether or not they achieved at least a 50 % reduction in the PHQ-9 or GAD-7 at 3 months relative to baseline. A logistic regression model will be built to predict whether or not they were a responder using age, gender, education, relationship status, household income, baseline belief in treatment credibility and outcome expectancy, and recruitment setting as predictors. We will also assess the contribution of BWW utilization, outpatient mental health visits, and medication changes during the intervention months as predictors of response.

Second, we will examine engagement with the BWW among ITG participants. The number of logins and total time on the site will be separately predicted with age, gender, education, relationship status, living situation, household income, baseline belief in treatment credibility and outcome expectancy, baseline PHQ-9 and GAD-7 scores, duration of current episode, recruitment setting and outpatient mental health visits.

##### Economic evaluation

The combination of program costs, out-of-pocket costs and costs in the health care system will be used to determine the total costs associated with participants in the program. Incremental cost-effectiveness will be assessed using an incremental cost-effectiveness ratio calculated as the difference in costs between DTG and ITG divided by the difference in outcomes between these groups. Confidence intervals around these estimates will be calculated using nonparametric bootstrapping resampling techniques (with 10,000 replications).

##### Power calculation

We have performed a power calculation assuming a sample size of 1000 participants which is the minimum target recruitment desired by the funder. Using data from other recent studies of internet based mental health interventions [10], we have calculated power assuming a conservative 30 % attrition rate. The minimal detectable difference between the two treatment groups was calculated assuming an ANCOVA analysis with a 0.8 correlation between baseline and 3-month follow-up RAS-r measurements. With a sample size of 700 accounting for attrition, allocated in a 2:1 ratio, using an alpha of 0.05 and power of 0.9, we are able to detect a difference of 1.35 on the RAS-r.

### Part 2. Qualitative process evaluation

#### Recruitment

Study participants. The qualitative research team will purposefully sample from the pool of interested ITG participants who consented to be contacted for an interview at study enrollment. We aim to interview 4-6 participants at each site, spanning different age categories, for a total of 12–15 patient participants.

##### Health care stakeholders

Qualitative interviews will be completed with 5–7 health care providers at each site; 2–4 organizational leaders at each site (for example, clinical managers); and 5–7 health system decision makers in Ontario. These participants will be identified by clinical site leads using a snowball sampling process to identify key informants.

#### Data collection

Qualitative interviews were developed with specific reference to Realist Evaluation methodology [26–28], incorporating a focus on the contexts, mechanisms, and outcomes of BWW implementation. Guided by the RE-AIM framework [25], the interviews will focus on different content for each participant group. Interviews with participants will focus on how the patient manages his or her mental health in the community, perspectives of BWW, and how BWW is used in daily life. If the participant has not engaged with BWW as evidenced by no or very few times logged in, the qualitative interview will include questions about why not, and what alternative strategies the patient uses to manage mental health in the community. Participants will be offered the opportunity to conduct the interviews remotely using personal computer videoconferencing with the option to enable screen sharing of the participant’s computer. Alternatively, a telephone interview will be arranged. Separate informed consent will be obtained for all qualitative interviews which will be audio-recorded.

Participants who opt in for the intervention extension arm of the study will again be asked whether they are interested in participating in a qualitative interview. Participants who identify as willing to be contacted may or may not have participated in a qualitative interview in the first phase of the study, and will be purposefully sampled and interviewed 2–4 weeks after the re-randomization process in the same manner as the initial interviews. We will again recruit 2–3 participants at each of the three sites for a total of 12–15 patient participants from the extension arm.

Qualitative interviews with health care provider participants will include questions regarding perspectives on mental health services in Ontario, and the value of virtual care interventions such as BWW for promoting self-management of mental health. Interviews with organizational leaders and health systems decision-makers will include questions about the context of mental health services in Ontario, the effectiveness of virtual care interventions to promote mental health self-management in the community, and the procurement and implementation of virtual care interventions such as BWW.

In addition to qualitative interviews, we will conduct qualitative observations of the BWW educational sessions delivered to health care providers by the implementation team from OTN. The researcher will take detailed notes regarding the content of the education session, including a specific focus on how health care providers react to the intervention, what questions they ask, and how the education session emphasizes implementation. These sessions will not be audio-recorded, rather observations will be guided by a structured observation worksheet.

#### Data analysis

Qualitative interviews and written observations will be transcribed into word documents and prepared for qualitative analysis. Both observational data and interview data will be analyzed using thematic analysis strategies [28, 41], identifying key themes that demonstrate important contextual influences and practices related to the implementation and evaluation of the virtual care application within the actual contexts of health care delivery. At least two researchers will review the transcripts, field and analytical notes independently and meet to develop a code workbook with emergent key themes. If a discrepancy emerges between the two researchers, a third researcher will review and consensus will be achieved. The findings of the qualitative data in conjunction with the quantitative outcomes data will be used to develop statements of the relationships between (a) key contextual factors, (b) the mechanisms by which they effect the implementation/uptake of BWW, and (c) the impact on the outcomes of BWW itself (in Realist Evaluation these statements are referred to as “Context-Mechanism-Outcome Configurations”) [28]. These statements will be used to develop understanding of (a) the specific mechanisms by which BWW is effective for users, and (b) strategies to inform the future implementation of BWW and/or similar interventions on a larger scale.

### Limitations

The requirements of health system stakeholders involved in this initiative necessitated a number of compromises in the design of this evaluation. First, the sample size requested by the funder necessitated broad inclusion criteria which is pragmatic but may present challenges around interpreting efficacy, particularly if no effect of the intervention is observed. We have limited recruitment to treatment settings where BWW is theorized to have the greatest applicability, and will seek to understand through our analysis and qualitative data if particular participant groups respond differently. Second, stakeholder desire for all participants to receive the intervention within a specific time interval meant that a control arm could not be continued throughout, and necessitated a short follow-up period, which will limit the interpretation of sustained intervention effectiveness. We have included a nested intervention extension arm open to interested participants to pragmatically evaluate the effectiveness of the sustained intervention in a self-selected patient group. Third, the specific intervention, BWW, was selected by the partners and although the process evaluation will seek to determine which components and mechanisms lead to the observed effects, it is uncertain how the findings of the study will be generalizable to other on-line mental health interventions.

## Discussion

Mental health recovery through self-management and life fulfillment is a priority across countries around the world in this recovery-oriented era [31]. Virtual strategies are a promising solution. Understanding the impact of these virtual strategies, such as BWW, on patient outcomes, patient experience, and health system costs is essential for informing whether and how health system decision-makers can support these strategies system-wide [42]. This requires clear evidence of effectiveness and an understanding of how the intervention works, for whom, and under what circumstances [28]. This study will produce such effectiveness data for BWW, while simultaneously exploring the characteristics and experiences of users for whom this and similar online interventions could be helpful, within our unique local context.

### Trial status

The trial is actively recruiting at all three sites. The first participant was recruited July 22, 2016 with a total of 475 participants consented to date.

## Abbreviations

AE: Adverse event
ANCOVA: Analysis of covariance
BWW: Big white wall
BWW: Big white wall
CIQ: Community integration questionnaire
CSRI: Client service receipt inventory
DSMB: Data safety and monitoring board
DTG: Delayed treatment group
ED: Emergency Department
EQ-5D-5 L: EuroQOL five dimensions questionnaire-5 level
GAD-7: Generalized anxiety disorder questionnaire-7 item
ICES: Institute for clinical evaluative sciences
ITG: Immediate treatment group
ITG-e: Immediate treatment group-extension
ITG-n: Immediate treatment group-no extension
OHIP: Ontario health insurance plan
OTN: Ontario telemedicine network
PHQ-9: Personal health questionnaire-9 item
RA: Research assistant
RAS-r: Recovery assessment scale-revised
RC: Research co-ordinator
RCT: Randomized controlled trial
